# Percutaneous coronary intervention in cardiac allograft vasculopathy: From lesion selection to longitudinal graft management

**DOI:** 10.1016/j.jhlto.2026.100677

**Published:** 2026-08-31

**Authors:** Ajay Dharan, Muhammed Faraz Ali, Nalin Dayawansa, Sarah Gutman, Hitesh Patel, David M. Kaye, James A. Shaw

**Affiliations:** a Department of Cardiology, Alfred Health, Melbourne, Victoria, Australia; b Department of Medicine, School of Translational Medicine, Monash University, Melbourne, Victoria, Australia; c Baker Heart and Diabetes Institute, Melbourne, Victoria, Australia

**Keywords:** cardiac allograft vasculopathy, percutaneous coronary intervention, coronary physiology, coronary microvascular dysfunction, heart transplantation

## Abstract

Cardiac allograft vasculopathy remains a major cause of late graft dysfunction and mortality after heart transplantation. Percutaneous coronary intervention can treat focal epicardial disease, but its role is complicated by diffuse epicardial involvement, microvascular dysfunction, donor-transmitted or conventional atherosclerosis, alloimmune injury, and limited transplant-specific interventional evidence. This narrative review examines cardiac allograft vasculopathy from the perspective of the interventional cardiologist, focusing on lesion selection, procedural optimization, and longitudinal management after intervention.

Longitudinal graft assessment contextualizes whether a stenosis reflects donor-derived disease, acquired atherosclerosis, progressive alloimmune vasculopathy, or overlapping processes, while donor-specific antibodies and rejection history provide complementary information regarding alloimmune risk. Coronary angiography remains central when revascularisation is contemplated, with intravascular imaging defining vessel dimensions, disease distribution, and procedural strategy. Coronary computed tomography angiography can exclude significant epicardial disease in appropriately selected recipients, whereas functional imaging and invasive coronary physiology characterize graft-wide haemodynamic and microvascular disease. However, no single anatomical, physiological, or immunological threshold has been prospectively validated for percutaneous coronary intervention.

Drug-eluting stents are generally favored for suitable focal lesions based on lower restenosis rates, although graft-level clinical benefit remains unproven. Post-intervention management should distinguish target-lesion failure from graft-wide disease progression and incorporate continued surveillance and transplant-directed disease modification. By distinguishing guideline-supported and transplant-specific evidence from native coronary extrapolation, this review proposes an evidence-informed interpretive framework in which percutaneous coronary intervention treats a mechanically correctable component of graft vascular disease rather than allograft vasculopathy itself.

## Background

Cardiac allograft vasculopathy (CAV) remains a major cause of late graft dysfunction and mortality after heart transplantation, reflecting interacting alloimmune and non-immune vascular injury that may manifest as diffuse concentric intimal proliferation, negative remodeling, focal epicardial stenoses, and microvascular dysfunction.^1^ Cardiac denervation further limits symptom-based recognition, such that clinically important disease is frequently identified through surveillance rather than typical angina.^1^, ^2^

These features complicate revascularisation. Percutaneous coronary intervention (PCI) is the main revascularisation strategy for anatomically suitable CAV, whereas coronary artery bypass grafting (CABG) is constrained by operative risk and diffuse or distal disease.^3^, ^4^ Observational data favor PCI for early procedural safety, but higher repeat revascularisation and treatment-selection bias preclude conclusions regarding comparative long-term efficacy.^3^ Heart transplant recipients are excluded from conventional PCI trials and appropriate-use frameworks, while transplant-specific evidence remains predominantly retrospective.^5^

The central interventional question is whether an angiographic stenosis represents a meaningful, mechanically treatable component of graft vascular disease. Longitudinal assessment, intravascular imaging, coronary physiology, and immunological data provide complementary context, but none have been prospectively validated as a standalone criterion for PCI selection.^1^, ^6^, ^7^, ^8^

Existing reviews comprehensively address the pathogenesis, diagnosis, and surveillance of CAV.^1^, ^2^, ^9^ This narrative review instead examines CAV from the perspective of the interventional cardiologist and addresses three practical questions: (1) does the identified lesion represent an appropriate target for PCI; (2) how should PCI be planned and optimized in the transplanted coronary circulation; and (3) how should the graft be managed and reassessed following intervention? We distinguish guideline-supported and transplant-specific evidence from extrapolation from native coronary intervention and propose an evidence-informed, pathophysiology-guided framework for clinical interpretation rather than a validated treatment algorithm.

## The longitudinal vascular substrate

Coronary disease in a heart-transplant recipient should be interpreted within the longitudinal history of the graft rather than from a single angiographic assessment. A stenosis considered for PCI may represent donor-transmitted atherosclerosis, conventional atherosclerosis developing after transplantation, progressive alloimmune CAV, or overlapping processes.^10^, ^11^ Although these substrates cannot always be distinguished in an individual lesion, their relative contribution provides important context for determining whether a focal stenosis represents a discrete mechanical target or one manifestation of progressive graft-wide vascular disease.

Pre-existing donor coronary disease is increasingly relevant with older donors and greater cardiovascular risk. ISHLT guidance states angiography at 4–6 weeks may be considered to identify donor disease, while IVUS with angiography at 4–6 weeks and approximately 1 year should be considered for early CAV assessment and prognostic information.^8^ Pre-transplant donor coronary assessment, where available, and early post-transplant imaging provide a baseline for subsequent interpretation.

Baseline lesions may reflect donor-transmitted plaque, whereas serial progression raises concern for acquired CAV; these processes can coexist, and morphology alone cannot establish aetiology.^8^, ^10^, ^11^ Donor plaque is often focal and eccentric, whereas CAV more often involves diffuse intimal proliferation, negative remodeling, distal disease, and microvascular dysfunction.^6^

These distinctions should inform expectations from revascularisation rather than create rigid treatment categories. A discrete flow-limiting lesion may remain amenable to PCI irrespective of presumed aetiology; however, focal donor-derived or conventional atherosclerosis may represent a greater proportion of the treatable disease burden, whereas progressive alloimmune, diffuse, or microvascular CAV implies greater residual graft-wide risk despite technically successful PCI.^1^, ^6^, ^8^

Immunological history provides complementary information. Donor-specific antibodies (DSA), particularly persistent Class II or complement-binding antibodies, and antibody-mediated rejection have been associated with CAV progression.^1^, ^12^, ^13^ In a cohort of 282 recipients, complement-binding de novo DSA were associated with severe CAV or mortality (adjusted HR 4.31), whereas non-complement-binding DSA were not.^13^ The relationship between DSA, antibody-mediated endothelial injury, and coronary microvascular dysfunction remains incompletely defined. Longitudinal DSA surveillance is guideline-supported, but no immunological marker has been validated to determine PCI candidacy.^5^, ^8^ Instead, ongoing alloimmune activity identifies a higher-risk biological substrate that may warrant parallel transplant-directed management even when a focal epicardial lesion is revascularised.^1^

Coronary calcium may complement assessment of conventional atherosclerotic burden but cannot exclude non-calcified or microvascular CAV.^14^ Associations between lipoprotein(a) and CAV are inconsistent; neither marker determines PCI candidacy.^15^, ^16^

Thus, longitudinal anatomy, disease progression, and immunological history should modify interpretation of the lesion rather than function as independent revascularisation criteria. The practical question remains whether the stenosis represents a meaningful focal target within the recipient’s broader graft vascular disease.

## From coronary lesion to PCI decision

Identification of coronary disease does not itself establish an indication for PCI. The interventional decision requires assessment of whether a discrete epicardial lesion is technically treatable and contributes meaningfully to coronary flow limitation, while recognizing disease that will remain outside the proposed target. Angiography, intravascular and non-invasive imaging, and coronary physiology provide complementary information; none is a validated standalone gatekeeper for PCI in CAV.^6^, ^8^

### Angiography and intravascular imaging

Invasive coronary angiography remains central when revascularisation is contemplated because it defines lesion location, vessel calibre, distal runoff, and the distribution of epicardial disease while permitting immediate physiological assessment, intravascular imaging, or PCI.^6^, ^8^ However, angiography depicts the lumen rather than the vessel wall. Diffuse concentric intimal proliferation and negative remodeling may therefore produce substantial CAV without a normal reference segment, underestimating disease severity and complicating lesion and stent sizing.^1^, ^6^ This discordance between angiographic luminal appearance and vessel-wall disease is illustrated in Figure 2.

**Figure 1:**
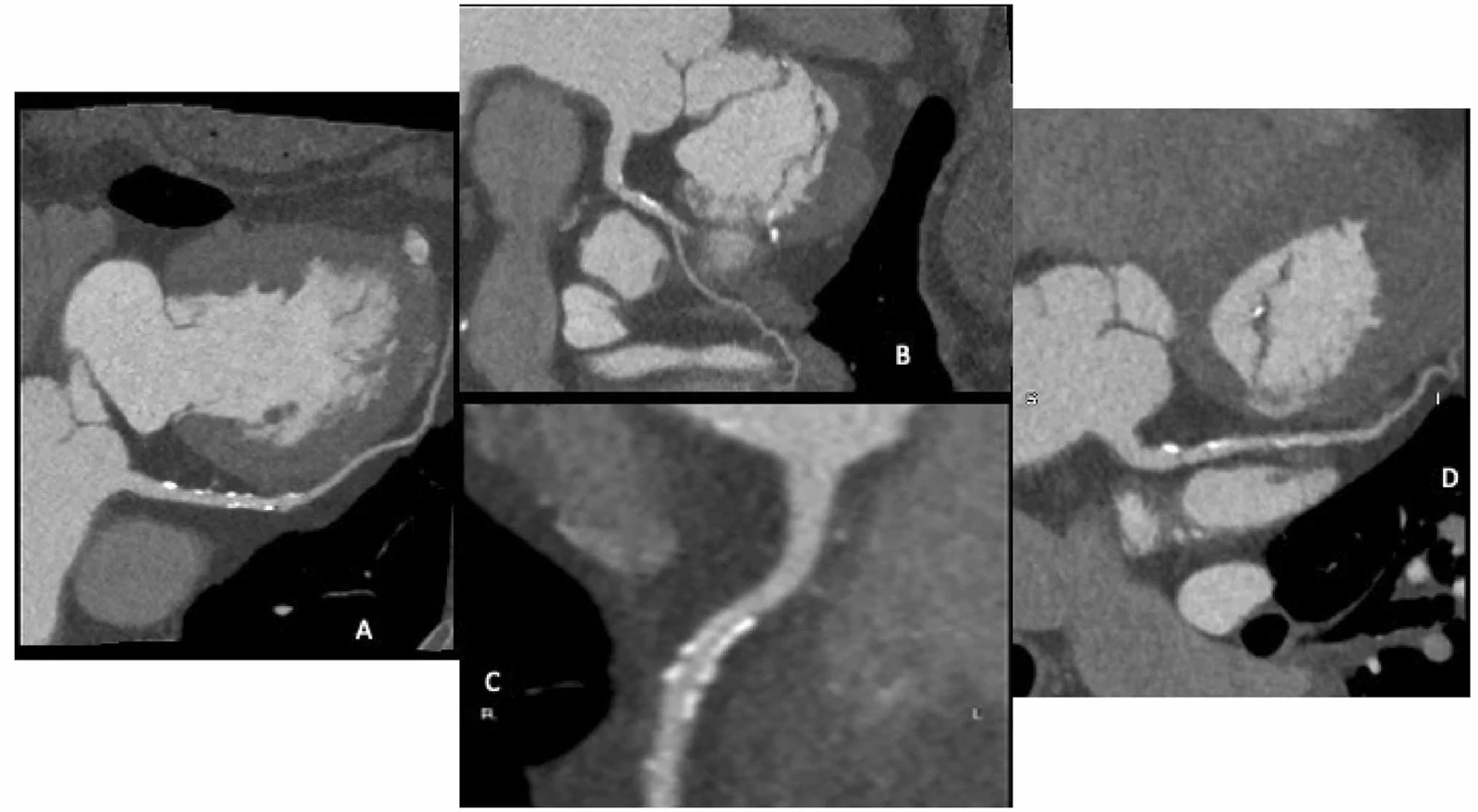
Coronary computed tomography angiography in cardiac allograft vasculopathy. Curved multiplanar reconstructions demonstrate diffuse coronary disease involving the left anterior descending artery (A), left circumflex artery (B), proximal right coronary artery (C), and first obtuse marginal branch (D), with calcified and non-calcified plaque and relative preservation of luminal calibre. The diffuse distribution illustrates the graft-wide epicardial disease that may coexist with or occur in the absence of a discrete target for focal revascularisation.

**Figure 2:**
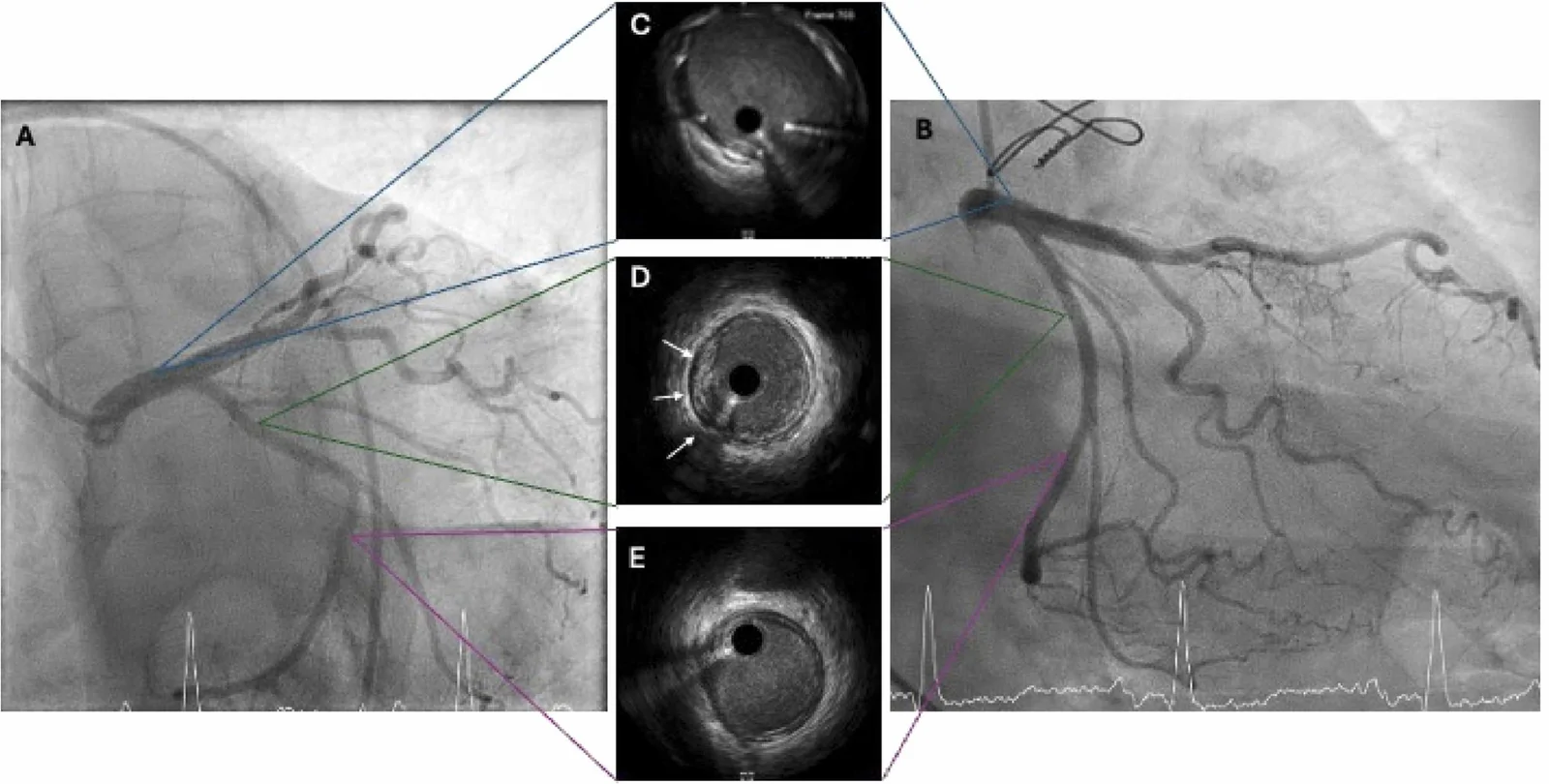
Angiographic–IVUS mismatch in cardiac allograft vasculopathy. Left anterior oblique caudal (A) and right anterior oblique caudal (B) projections show a patent left main-to-left anterior descending stent without angiographically visible stenosis. IVUS pullback of the left circumflex artery demonstrates a patent left main stent with minimal neointima (C), diffuse hyperechoic, low-attenuation plaque in the proximal-to-mid left circumflex consistent with cardiac allograft vasculopathy (D, arrows), and a relatively preserved distal reference segment (E). The discordance illustrates how angiography may underestimate diffuse vessel-wall disease and the complementary role of intravascular imaging when defining the substrate and extent of disease relevant to PCI. IVUS, Intravascular ultrasound; PCI, Percutaneous coronary intervention.

Intravascular ultrasound (IVUS) and optical coherence tomography (OCT) can clarify lesion length, vessel dimensions, intimal or plaque distribution, calcification, and proximal and distal landing zones. ISHLT guidance supports IVUS and OCT as adjuncts rather than mandating routine use.^8^ Although randomized trials in native coronary disease have demonstrated improved outcomes with intravascular imaging-guided PCI, there are no transplant-specific randomized trials evaluating this strategy.^6^, ^17^ Nevertheless, intravascular imaging is particularly valuable when vessel dimensions are uncertain, disease is long or diffuse, or restenosis has occurred.^6^

### Non-invasive imaging

Computed Tomography Coronary Angiography (CTCA) can reliably exclude significant epicardial CAV in appropriately selected recipients and can demonstrate the distribution and morphology of epicardial disease (Figure 1). A meta-analysis of 13 prospective studies including 615 heart-transplant recipients reported sensitivity of 94%, specificity of 92%, negative predictive value of 99%, and positive predictive value of 67% for significant CAV.^18^ Accordingly, ISHLT guidance supports CTCA as a non-invasive alternative to coronary angiography for surveillance of appropriately sized epicardial vessels.^8^ Elevated heart rate, small distal vessels, calcification, and coronary stents remain limitations.^19^ For PCI assessment, CTCA can identify or help exclude significant epicardial disease but is not a standalone revascularisation gatekeeper.

Functional imaging assesses the haemodynamic consequences of graft vascular disease. Positron Emission Tomography (PET)-derived myocardial blood flow and flow reserve provide diagnostic and prognostic information.^8^, ^20^ In 344 recipients, lower PET CAV grades had negative predictive values of 0.93–0.95 for subsequent moderate-to-severe angiographic CAV, whereas PET CAV grade 2/3 was associated with increased mortality (HR 2.86).^20^ Stress cardiac magnetic resonance (CMR) is an alternative perfusion modality, whereas dobutamine echocardiography has limited sensitivity.^8^, ^21^ Functional imaging defines graft-wide burden but cannot identify a focal stenosis as the treatable culprit.^8^, ^22^

### Invasive coronary physiology

Fractional Flow Reserve (FFR), Coronary Flow Reserve (CFR), and Index of Microcirculatory Resistance (IMR) interrogate different components of graft vascular dysfunction.^23^, ^24^ FFR assesses pressure loss attributable to epicardial disease and may help determine the significance of an intermediate focal stenosis.^24^ However, the conventional FFR threshold of ≤0.80 derives predominantly from native coronary disease, and no transplant-specific randomized trials have evaluated FFR-guided PCI.^23^, ^24^

CFR reflects integrated epicardial and microvascular vasodilatory capacity, whereas IMR more specifically assesses microvascular resistance.^7^, ^25^, ^26^ ISHLT guidelines recognize coronary flow physiology alongside angiography as useful for detecting small-vessel disease.^8^ Observational transplant studies associate impaired CFR and elevated IMR with rejection, CAV progression, and adverse outcomes, but reported IMR thresholds vary according to timing and endpoint, from approximately 15 to ≥25.^7^, ^26^, ^27^ No unified IMR threshold has been validated for PCI selection.

The value of combined physiology is therefore descriptive rather than prescriptive. A discrete stenosis associated with focal epicardial pressure loss represents a mechanistically plausible PCI target.^23^, ^24^ When impaired CFR or elevated IMR coexists, PCI may still correct the epicardial component, but diffuse or microvascular disease will remain. Conversely, abnormal microvascular physiology without a meaningful epicardial stenosis does not provide an indication for PCI.^8^, ^24^

**Figure 3:**
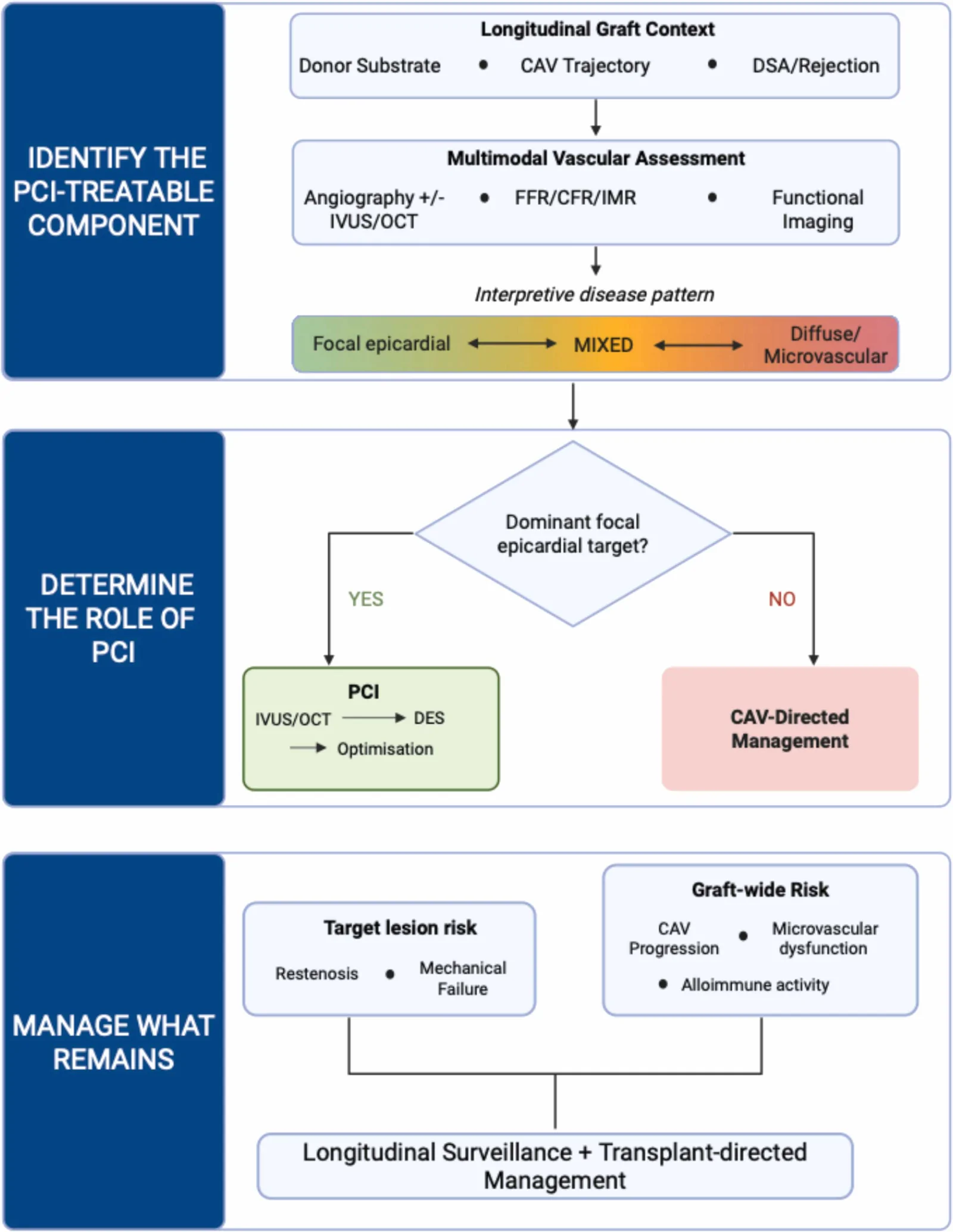
Longitudinal, pathophysiology-informed framework for percutaneous coronary intervention in cardiac allograft vasculopathy. Longitudinal graft context and multimodal vascular assessment are integrated to identify a potentially PCI-treatable focal epicardial component and contextualize residual diffuse, microvascular, and alloimmune disease. Following PCI, target-lesion risk is distinguished from graft-wide CAV progression requiring longitudinal surveillance and transplant-directed management. Disease patterns are interpretive constructs rather than validated phenotypes, and the framework is not a validated treatment algorithm. CAV, cardiac allograft vasculopathy; CFR, coronary flow reserve; DES, drug-eluting stent; DSA, donor-specific antibody; FFR, fractional flow reserve; IMR, index of microcirculatory resistance; IVUS, intravascular ultrasound; OCT, optical coherence tomography; PCI, percutaneous coronary intervention.

These findings can be conceptualized as overlapping atherosclerotic-predominant, focal epicardial-predominant, diffuse/microvascular-predominant, and mixed patterns. These are interpretive constructs rather than validated diagnostic phenotypes; their purpose is not to assign treatment, but to clarify what proportion of graft vascular disease can plausibly be addressed by focal revascularisation.^1^, ^8^ Integrating longitudinal graft context with multimodal vascular assessment therefore provides a practical framework for identifying a potentially PCI-treatable component, determining the role of focal intervention, and anticipating residual target-lesion and graft-wide risk (Figure 3).

## PCI execution and longitudinal management

Once a meaningful epicardial target has been identified, PCI should aim for a durable focal result while recognizing that disease elsewhere in the graft remains untreated. Focal proximal or mid-vessel stenoses in adequately sized arteries are generally more amenable to intervention than long diffuse lesions, severe distal disease, or progressive small-vessel CAV.^6^, ^8^, ^28^ CAV may additionally present with long lesions, small-calibre vessels, negative remodeling, and diseased angiographic reference segments, all of which can complicate lesion assessment and increase the likelihood of incomplete revascularisation or restenosis.^6^, ^8^ Lesion length, vessel calibre, reference-segment quality, distal runoff, and the burden and distribution of disease elsewhere should therefore inform whether durable revascularisation is feasible.^8^ Conversely, advanced diffuse CAV accompanied by progressive graft dysfunction may shift management away from focal revascularisation towards advanced heart failure therapy or consideration of re-transplantation.^8^, ^28^

### Procedural optimization and restenosis

Diffuse intimal proliferation and negative remodeling can make angiographic reference-vessel selection unreliable.^6^ IVUS or OCT can assist with vessel sizing, lesion length, and landing-zone selection, while post-PCI imaging can identify under-expansion, malapposition, edge disease, or other potentially correctable mechanisms of failure.^6^, ^29^ This may be particularly relevant in CAV, where apparently normal angiographic reference segments may themselves contain substantial intimal disease.^6^ Although randomized native-coronary trials support intravascular imaging-guided PCI, no comparable randomized evidence exists in heart-transplant recipients.^29^ Calcified, bifurcation, and other complex lesions should otherwise be treated according to contemporary native-PCI principles, as transplant-specific evidence for atherectomy, intravascular lithotripsy, or dedicated complex-PCI strategies remain limited.^30^

Drug-eluting stents (DES) are generally preferred to bare-metal stents for focal CAV. Earlier transplant studies consistently showed lower in-stent restenosis (ISR) with DES, including rates of 12% versus 30% with BMS in one comparative cohort.^31^, ^32^ More recent observational data support the use of newer-generation DES, although restenosis remains relevant, particularly in small-calibre vessels.^33^, ^34^ A recent single-center study of 157 analysed recipients found similar ISR, target-lesion revascularisation, and death/re-transplantation with biodegradable- versus durable-polymer everolimus-eluting stents, although target-vessel non-target-lesion revascularisation was lower with biodegradable-polymer DES.^35^

Recurrent stenosis should prompt assessment of mechanism, ideally with intravascular imaging, to distinguish stent under-expansion, edge disease, or other mechanical factors from recurrent neointimal proliferation and progression of disease adjacent to the treated segment.^6^ Importantly, restenosis and progression in remote non-intervened coronary segments may occur in parallel, reinforcing the distinction between target-lesion failure and graft-wide disease progression.^36^ Repeat PCI should therefore be tailored to the mechanism identified rather than assumed to represent uniform biological restenosis. Drug-coated balloons may avoid additional stent layers in selected cases of restenosis and have an established evidence base in native-coronary practice, but transplant-specific experience remains limited to small series and case-level evidence.^37^, ^38^, ^39^

### Systemic therapy after PCI

Statin therapy remains fundamental because PCI does not address the systemic vascular substrate. Randomized pravastatin data demonstrated improved 10-year survival (68% vs 48%) and freedom from CAV or death (43% vs 20%), while subsequent meta-analytic evidence supports reduction in mortality, rejection, and CAV.^40^, ^41^ Statins are therefore recommended for adult heart-transplant recipients irrespective of baseline cholesterol.

No transplant-specific evidence-derived LDL-C target has been established. ISHLT guidelines consider an LDL-C target below 100 mg/dl (2.5 mmol/liter) reasonable for most recipients, with more aggressive lowering in established CAV.^8^ However, recent randomized trials of evolocumab and alirocumab achieved substantial LDL-C reduction without significantly attenuating IVUS-defined CAV progression.^42^, ^43^ These findings reinforce the multifactorial biology of CAV rather than arguing against intensive lipid management when otherwise indicated.

The transplant-specific significance of lipoprotein(a) also remains uncertain. Elevated Lp(a) was associated with moderate-to-severe CAV in one observational cohort, whereas a subsequent multicentre cohort of 385 recipients found no association between Lp(a) ≥ 50 mg/dl and clinically significant CAV.^15^, ^16^ No transplant-specific Lp(a) threshold or targeted treatment strategy has therefore been established.

Immunosuppressive modification, including the consideration of proliferation-signal inhibitor-based strategies in appropriate recipients with CAV, remains transplant-directed disease modification and should be individualized according to the broader immunological and clinical context rather than PCI itself.^8^, ^44^

Antiplatelet therapy is extrapolated from non-transplant PCI, with no transplant-specific evidence supporting a particular DAPT duration or P2Y12 inhibitor. Treatment should balance ischemic risk against bleeding, renal dysfunction, concomitant anticoagulation, and anticipated invasive procedures; pharmacogenetic P2Y12 selection remains untested in transplant recipients.^8^, ^30^

### Surveillance after PCI

Post-PCI assessment should distinguish target-lesion failure from progression of graft-wide CAV. Revascularisation in another coronary territory reflects disease progression rather than failure of an initially durable PCI, whereas recurrent target-lesion stenosis should prompt evaluation for a correctable mechanical mechanism.^6^, ^36^

ISHLT guidelines recommend follow-up coronary angiography at 6 months after PCI to evaluate in-stent restenosis and progression of CAV.^8^ Beyond this time point, no prospectively validated post-PCI surveillance schedule has been established. Recipients should remain within established CAV surveillance pathways, with invasive reassessment guided by graft dysfunction, suspected ischemia, recurrent restenosis, rapid disease progression, or concerning non-invasive findings.^8^ CTCA may assess untreated epicardial segments, while PET-derived myocardial blood flow and flow reserve can provide longitudinal information regarding graft-wide vascular dysfunction.^19^ Emerging PET data suggest that reassuring studies may support longer intervals between invasive assessments in selected recipients, although this has not been prospectively validated following PCI.^20^

Future studies should distinguish procedural and anatomical endpoints (target-lesion failure, target-vessel progression, and non-target CAV progression) from graft-level clinical outcomes including graft dysfunction, death and re-transplantation, while integrating lesion morphology, intravascular imaging, physiology, and immunological status. Such studies are required to determine whether contemporary PCI improves outcomes beyond local lesion durability.

## Conclusion

PCI in CAV should be considered treatment of a mechanically correctable component of graft vascular disease rather than treatment of CAV itself. Lesion selection should integrate longitudinal graft context with anatomical and physiological assessment while recognizing residual diffuse, microvascular, and alloimmune disease.^1^, ^6^, ^13^, ^24^ No imaging, physiological, or immunological threshold has been prospectively validated for PCI selection; the proposed framework is therefore an evidence-informed interpretive model rather than a treatment algorithm. Prospective multicentre studies integrating lesion anatomy, physiology, imaging, immunological status, and longitudinal outcomes are required to determine whether this approach improves PCI selection and long-term graft outcomes (Table 1).

**Table 1:** Evidence Status and Role of Contemporary Assessments and Therapies in PCI for Cardiac Allograft Vasculopathy

**Component**	**Guideline position**	**Transplant evidence**	**PCI relevance**
Baseline angiography±IVUS	Angiography at 4–6 weeks may be considered; IVUS with angiography at 4–6 weeks and approximately 1 year should be considered for early CAV assessment	Longitudinal studies define donor disease and intimal progression	Establishes baseline substrate and disease trajectory
CTCA	Non-invasive alternative to angiography in suitable ≥2-mm epicardial vessels	13 studies, *n* = 615: sensitivity 94%, specificity 92%, NPV 99%	Helps exclude significant epicardial CAV
PET/stress imaging	Stress CMR may be considered; other modalities individualized	Diagnostic/prognostic cohorts; strongest quantitative evidence for PET	Defines graft-wide haemodynamic burden; not lesion-specific
FFR	No CAV-specific FFR-guided PCI recommendation	Observational data; ≤0.80 extrapolated from native CAD	Assesses focal epicardial significance; no validated CAV PCI threshold
CFR/IMR	May complement angiography for small-vessel disease	Associated with rejection, CAV progression, and adverse outcomes; thresholds heterogeneous	Defines diffuse/microvascular burden; no PCI-selection cut-off
DSA/rejection	Longitudinal surveillance guideline-supported	Persistent Class II and complement-binding DSA associated with adverse CAV outcomes	Defines alloimmune risk; not a PCI-selection biomarker
DES versus BMS	No transplant-specific device recommendation based on randomized evidence	DES associated with lower ISR; graft-level clinical benefit unproven	Supports DES for suitable focal lesions; graft-wide benefit unproven
Imaging-guided PCI	Not specifically mandated after transplantation	Limited transplant data; strong native-PCI evidence	Supports sizing, landing-zone selection, optimization, and ISR assessment
Drug-coated balloon	No transplant-specific recommendation	Limited transplant evidence; extrapolated from native ISR	Selected ISR option; transplant-specific efficacy uncertain
Statin/lipid therapy	Statins recommended; LDL-C <100 mg/dl (2.5 mmol/liter) is reasonable, with more intensive lowering considered in established CAV	Randomized and meta-analytic evidence supports statins	Graft-wide risk modification; independent of PCI selection
Immunosuppressive modification	PSI-based strategies should be considered in appropriate recipients	Sirolimus/everolimus strategies attenuate CAV progression	Graft-wide disease modification; not a PCI criterion
Post-PCI surveillance	Angiography at approximately 6 months after PCI is recommended because of high restenosis rates	ISR and progression in untreated territories documented	Separates target-lesion failure from graft-wide CAV progression

BMS, bare-metal stent; CAD, coronary artery disease; CAV, cardiac allograft vasculopathy; CTCA, coronary computed tomography angiography; CFR, coronary flow reserve; CMR, cardiovascular magnetic resonance; DES, drug-eluting stent; DSA, donor-specific antibody; FFR, fractional flow reserve; IMR, index of microcirculatory resistance; ISR, in-stent restenosis; IVUS, intravascular ultrasound; LDL-C, low-density lipoprotein cholesterol; NPV, negative predictive value; PCI, percutaneous coronary intervention; PET, positron emission tomography; PSI, proliferation-signal inhibitor.

Guideline position reflects current heart-transplant guidance where available. Transplant evidence includes randomized and observational studies specific to heart-transplant recipients; where transplant-specific evidence is limited, relevant evidence is extrapolated from native coronary disease.^6^, ^8^, ^10^, ^12^, ^13^, ^18^, ^19^, ^20^, ^24^, ^25^, ^35^, ^37^, ^41^, ^44^

## Declaration of Competing Interest

The authors declare that they have no known competing financial interests or personal relationships that could have appeared to influence the work reported in this paper.
